# Declining Trends of Heart Rate Variability According to Aging in Healthy Asian Adults

**DOI:** 10.3389/fnagi.2020.610626

**Published:** 2020-11-26

**Authors:** Jungmi Choi, Wonseok Cha, Min-Goo Park

**Affiliations:** ^1^Human Anti-Aging Standards Research Institute, Uiryeong-gun, South Korea; ^2^Department of Plant Quarantine, Animal and Plant Quarantine Agency (APQA), Gimcheon-si, South Korea; ^3^Institute of Agriculture and Life Science, Gyeongsang National University, Jinju-si, South Korea

**Keywords:** aging index, heart rate variability, declining trends, autonomic nervous system, healthy Asians

## Abstract

Heart rate variability (HRV) indices correlate with aging and are related to the autonomic nervous system. However, the trend of HRV with age has not been explored for the Asian population. Therefore, we proposed a linear regression model of HRV indices that decreased with aging in healthy Asian adults. HRV parameters [High frequency (HF), Low frequency (LF), Very low frequency (VLF), Total power (TP), HRV triangular index (HRV-index), Standard deviation of the normal-to-normal interval (SDNN), and Proportion of normal-to-normal intervals greater than 50 ms (pNN50)] were measured in a total of 300 healthy participants (150 men and 150 women) aged 19–69 years stratified into five age groups: 19–29, 30–39, 40–49, 50–59, and 60–69 years comprising 60 people each in Seoul, South Korea. A simple regression analysis was performed to reveal the linear declining trend of HRV indices with age. Independent *t*-tests were conducted to investigate the gender differences in HRV values depending on each age group. The values of all HRV indices showed a decreasing trend with age in healthy Korean adults, as observed in the Western population (*P* < 0.001 for all indices); HF (*Y* = −0.039x + 6.833, *R*^2^ = 0.287), LF (*Y* = −0.047x + 7.197, *R*^2^ = 0.414), VLF (*Y* = −0.025x + 6.861, *R*^2^ = 0.177), TP (*Y* = −0.034x + 8.082, *R*^2^ = 0.352), HRV-index (*Y* = −0.125x + 15.628, *R*^2^ = 0.298), SDNN (*Y* = −0.502x + 53.907, *R*^2^ = 0.343), and pNN50 (*Y* = −0.650x + 53.852, *R*^2^ = 0.345) all decreased with age. There was no significant gender difference in any HRV parameter. A linear regression model of various HRV indices has been presented considering the age of healthy Asians, which may be useful to prevent diseases related to the autonomic nervous system by estimating or tracking autonomic functional degeneration in the Asian population.

## Introduction

The aging of the population is rapidly progressing in many countries worldwide and is becoming a significant challenge to public health care (Lunenfeld and Stratton, 2013). Therefore, it is necessary to identify reliable factors that reflect healthy aging. The autonomic nervous system (ANS) is one of the key regulatory systems affected by aging, which may contribute to cognitive impairments and neuropsychiatric symptoms (Pfeifer et al., 1983; Bishop et al., 2010; Simpson et al., 2014; Choi et al., 2019).

Heart rate variability (HRV) has been used primarily to evaluate the overall autonomic function in clinical practice. HRV allows for the assessment of autonomic cardiovascular function and wellness as well as the monitoring of sleep, stress, and fatigue (Kleiger et al., 2005; Maunder et al., 2006; Baek et al., 2009; Patel et al., 2011; Gouin et al., 2013). HRV can be investigated non-invasively using a photoplethysmogram (PPG), which can detect blood volume changes in the microvascular bed of the tissue as well as using electrocardiograph (ECG) findings (Task Force of The European Society of Cardiology, and The North American Electrophysiology Society of Pacing and Electrophysiology, 1996; Agelink et al., 2001; Allen, 2007; Liang et al., 2018; Elgendi et al., 2019). The recommended measurement length for HRV is 24 h for long-term monitoring and 5 min for short-term monitoring (Task Force of The European Society of Cardiology, and The North American Electrophysiology Society of Pacing and Electrophysiology, 1996). A patient’s physiological regulation can be monitored with 24-h HRV analysis, but this is difficult and not very practical. Short-term monitoring provides a test result almost immediately. It is therefore suitable for outpatient monitoring and for situations wherein results are urgently needed (Baek et al., 2015). Recently, the use of ultra-short HRV analysis based on recordings shorter than 5 min has also been studied to increase the applicability of HRV analysis according to the development of wearable devices for monitoring public health (Baek et al., 2015; Pecchia et al., 2018; Landreani et al., 2019).

In particular, HRV indices feature distinct aging trends that increase or decrease linearly with advancing age, which are useful for assessing functional degradation (weakness) of autonomic nerves (Reardon and Malik, 1996; Task Force of The European Society of Cardiology, and The North American Electrophysiology Society of Pacing and Electrophysiology, 1996; Dietrich et al., 2006; De Meersman and Stein, 2007; Voss et al., 2015). In 2019, HRV was suggested as a useful marker for disease prevention in the elderly (Tan et al., 2019). The authors of this study have also shown that the HRV indices, which were measured with the same instruments and protocols as those in this study, significantly increased after therapy in forest, resulting in a positive effect (Yi et al., 2019).

Studies on the aging trends of HRV have been conducted mainly in the Western population (Reardon and Malik, 1996; Task Force of The European Society of Cardiology, and The North American Electrophysiology Society of Pacing and Electrophysiology, 1996; Dietrich et al., 2006; De Meersman and Stein, 2007; Voss et al., 2015). Thus, the age-related trend of HRV in Asian adults also needs to be explored. Only a few studies have been performed, but it was difficult to quantitatively estimate the functional age for each HRV index because the number of people by age group was quite different, which was attributed to the different purposes of the studies, such as assessing the reliability of ultra-short HRV analysis or the primary effect of aging on HRV. It was found that the trend model for most HRV indices was not specifically presented (Fukusaki et al., 2000; Baek et al., 2015).

Therefore, we proposed a linear regression model of HRV indices that decreased with age in healthy Asian adults by having a uniform number of subjects stratified by gender and age. Lack of reliability in the ultra-short-term HRV analysis of less than 5 min has been raised; thus, only 5-min measurement data were used in the study (Pecchia et al., 2018).

## Methods

### Subjects

A total of 300 healthy participants (150 men and women each) aged 19–69 years (stratified into five age groups: 19–29, 30–39, 40–49, 50–59, and 60–69 years comprising 60 people each) were recruited at the Clinical Trial Center of Asan Medical Center (AMC), Seoul, South Korea. Subjects were selected according to the following criteria for inclusion:

(1) Healthy Korean men and women aged 19–69.
(2) Non-smokers.
(3) Non-pregnant woman.
(4) Persons with a body mass index of 18–30 kg/m^2^ (body mass index calculation method: weight [(kg)/height (m)^2^].
(5) Persons who voluntarily decided to participate in this clinical trial for 2–3 h and agreed in writing.

However, the people with the following conditions were excluded from the study:

(1) Person who ate foods other than water within 1 h of the pulse measurements.
(2) Persons who performed strenuous exercise within an hour of the pulse measurements.
(3) Persons who were judged to be lacking in sleeping by less than 4 h in the previous day.
(4) Persons who were expected to have many hand movements because they could not control their movement during the pulse measurements.
(5) Persons who were currently taking medication for the purpose of treating the central nervous system or cardiovascular disease.
(6) Persons who were taking medication for the treatment of hyperthyroidism or hypothyroidism.
(7) Persons who had been diagnosed with diabetes for more than 10 years.

This observational study was conducted as part of the statistical standardization of quantitative PPG parameters for healthy Koreans, which is a brain-frontier project carried out by the Ministry of Education. Written informed consent was obtained from each subject prior to study participation. The Institutional Review Board (ASAN MEDICAL CENTER INSTITUTIONAL REVIEW BOARD, IRB number: AMC-IRB-2007-0305) approved the study protocol. After basic demographic information was collected, the participants were examined with PPG by registered clinical research nurses. The study was performed in accordance with the Declaration of Helsinki guidelines.

### Heart Rate Variability Measurements

All subjects were seated in a relaxed state with their eyes open, and HRV was measured for 5 min with photoplethysmography (PPG). A PPG sensor [Model: ubpulse T1 (Pulse Analyzer, KFDA Certification No. 11-1296), LAXTHA Inc., South Korea] was applied to the left index finger; PPG sensors are comparable to electrocardiograms in the quality of their Heart Rate Tachograms (Jeyhani et al., 2015).

The hands attached to the sensors were placed on the table at heart level and relaxed, and if there were manicured fingernails or alien substances on the fingernails of the finger attached to the pulse electrode, they were removed. Also removed were articles that put pressure on the arms or fingers, such as tight-fitting sleeves, disposable bands and rubber bands, and the subjects were cautioned not to take deep or abdominal breathes during the measurement to prevent respiratory sinus arrhythmia (RSA) (Hirsch and Bishop, 2017).

**FIGURE 1:**
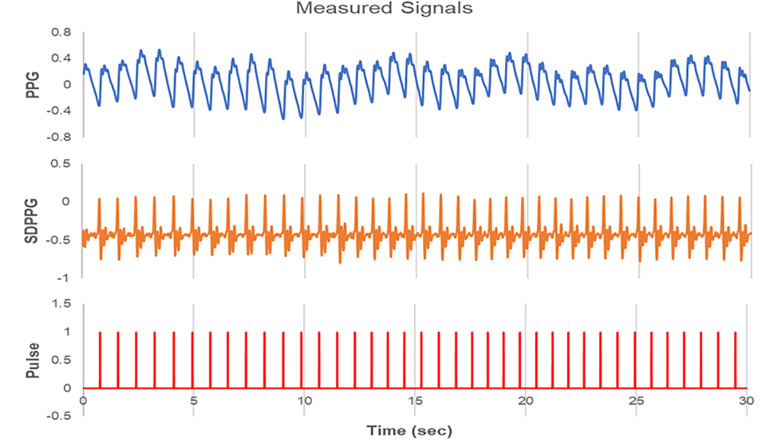
Signals measured by the PPG device (ubpulse T1). Photoplethysmogram (PPG), second derivative PPG (SDPPG), and pulse signals were measured simultaneously. PPG is expressed as a waveform detected the blood volume at the fingertip. SDPPG is illustrated by the second derivation of the PPG. The pulse signal has a value of 1 only at the maximum positive peak position in each SDPPG and a value of 0 the rest of the time.

With the device, the PPG, second derivative PPG (SDPPG), and pulse signals were measured simultaneously, as shown in Figure 1. The pulse signal has a value of 1 only at the maximum positive peak position in each SDPPG waveform and a value of 0 the rest of the time. The operator minimized data corruption by monitoring the signal and assured that it is not distorted by finger movements or shaking of the sensor. All HRV metrics were measured at a relatively constant time zone (9 A.M. to 6 P.M.) to avoid circadian variation depending on subjects (Bonnemeier et al., 2003).

### Biomarkers and Calculations of Heart Rate Variability

The HRV indices used in this study were HF, LF, VLF, TP, HRV-index, SDNN and pNN50, and the analysis was conducted based on international standard methods (Task Force of The European Society of Cardiology, and The North American Electrophysiology Society of Pacing and Electrophysiology, 1996).

Simple time-domain variables that can be calculated include the mean NN interval (heart period, HP), the mean heart rate (HR) and the standard deviation of the NN interval (SDNN), i.e., the square root of the variance. Since variance is mathematically equal to the total power of spectral analysis, SDNN reflects all the cyclic components responsible for variability in the period of recording. The most commonly used measures derived from interval differences include NN50, the number of interval differences of successive NN intervals greater than 50 ms, and pNN50, the proportion (%) derived by dividing NN50 by the total number of NN intervals.

The series of NN intervals can also be converted into a geometric pattern, such as the normalized sample density distribution of NN interval durations, and a simple formula is used that judges the variability based on the geometric and/or graphical properties of the resulting pattern. Most geometric methods require the NN interval sequence to be measured on or converted to a discrete scale that is not too fine or too coarse, which permits the construction of smoothed histograms. Most researched applications use bins approximately 8 ms long.

The HRV triangular index (HRV-Index) is the integral of the density distribution (i.e., the number of all NN intervals) divided by the maximum of the density distribution. Using a measurement of NN intervals on a discrete scale and the normalized distribution with the sum over bins of 1, the HRV-Index is approximated as follows:

HRV-Index = 1/(maximum of the normalized density distribution).

The HRV-index is an index that quantifies the sharpness and flatness of the geometric features and is known as an important indicator of cardiorespiratory performance. The larger the HRV-index and the lower and wider the distribution pattern, the healthier the person is. The major advantage of geometric methods lies in their relative insensitivity to the analytical quality of the series of NN intervals (Malik et al., 1993). The major disadvantage is the need for a reasonable number of NN intervals to construct the geometric pattern.

Various spectral methods for the analysis of the heart period variability have been applied since the late 1960s (Kay and Marple, 1981). Power spectral density (PSD) analysis provides the basic information of how power (i.e., variance) is distributed as a function of frequency. Methods for the calculation of PSD may be generally classified as non-parametric and parametric. The advantages of the non-parametric methods are: (a) the simplicity of the algorithm employed [Fast Fourier Transform (FFT) in most cases] and (b) the high processing speed. In this study, the PSD of heart period variability was calculated by the FFT-based non-parametric algorithm without any spectral window. Three main spectral components are distinguished in a spectrum calculated from short-term recordings of 2 to 5 min (Saykrs, 1973; Akselrod et al., 1981; Pagani et al., 1986; Malliani et al., 1991; Hirsch and Bishop, 2017): very low frequency (VLF), low frequency (LF), and high frequency (HF) components. Measurement of VLF, LF, and HF power components is usually made in absolute values of power (ms^2^). The high frequency (HF) range is 0.15–0.4 Hz, which is a relatively fast rhythm range and reflects HF activity in the ANS. The low frequency (LF) range is 0.04–0.15 Hz, which is a relatively low frequency range, and reflects LF activity. The lower frequency range of 0.003–0.04 is a very low frequency range (VLF). The sum of the powers of all the regions is referred to as the Total Power (TP). These power indices are generally rescaled by the natural logarithm for a normal distribution. About all HRV indices, the higher the indices indicate the younger and healthier ANS.

Ectopic beats, arrhythmic events, missing data, and noise effects may alter the estimation of the PSD of HRV. Proper interpolation (or linear regression or similar algorithms) of preceding/successive beats to the HRV signals or its autocorrelation function may reduce this error. Preferentially, short-term recordings that are free of ectopy, missing data, and noise should be used. Therefore, in our study, clean pulse data were selected and used for the determination of HRV indices.

### Statistical Analysis

Simple regression analysis was performed to reveal the monotonically decreasing trend of HRV indices according to the age of the subjects. The independent *t*-test was conducted to analyze the gender differences with respect to HRV parameters in each age group. Statistical analysis was performed using SPSS ver. 23 (SPSS Inc., Chicago, IL, United States, 2009). The significance level was set to α = 0.05 for all statistical tests (two-tailed).

## Results

**FIGURE 2:**
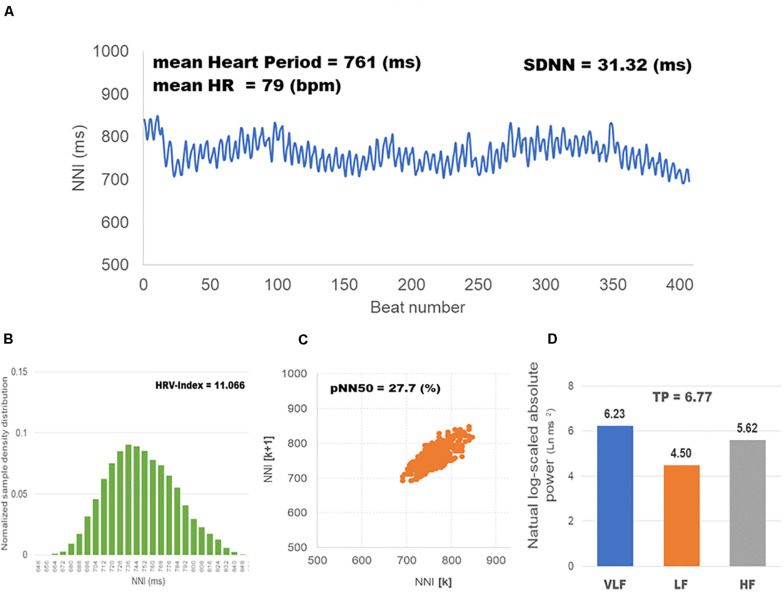
HRV indices calculated from the pulse time series measured for one participant. HF, LF, and VLF were expressed as the natural log-scaled power (ms^2^) of each frequency range; HR as beats per minute (bpm); SDNN as the standard deviation (ms) of heart rate variability; and pNN50 as the proportion (%) derived by dividing NN50 by the total number of NN intervals. Only HRV-index is unitless. **(A)** **Heart period variability**, the change in the interval between heartbeats; **(B) Normalized sample density distribution**, the integral of the density distribution (i.e., the number of all NN intervals) divided by the maximum of the density distribution; **(C) Phase plot**, the proportion of the number of successive NN intervals greater than 50 ms in the total number of NN intervals; **(D) Spectral power indices**, the heart period variability is decomposed into HF, LF, VLF frequency components.

A total of 300 subjects were recruited for this study. Before analysis, two experts screened the dataset and eliminated inappropriate samples that could not be analyzed owing to measurement errors by finger movements or shaking of the sensor. Therefore, the final number of participants remaining for analysis was 291 (144 men and 147 women), as shown in Table 1. Figure 2 shows HRV indices calculated from the pulse time series measured for one subject.

**TABLE 1:** Comparison of HRV indices between male and female subjects in each age group.

	20 years		30 years		40 years		50 years		60 years		Total	
Variables	Male	Female	Male	Female	Male	Female	Male	Female	Male	Female	Male	Female
Subjects, n	27	29	28	31	30	30	29	29	30	28	144	147
Age, y	24.3 ± 2.6	23.5 ± 3.2	36.4 ± 2.6	35.2 ± 3.0	44.4 ± 2.9	45.1 ± 3.0	54.3 ± 2.8	52.4 ± 2.1**	63.1 ± 2.4	64.0 ± 2.6	44.8 ± 13.9	43.8 ± 14.1
HF, (ms)^2^	5.72 ± 0.74	6.04 ± 0.75	5.47 ± 0.77	5.57 ± 0.63	4.96 ± 0.81	5.04 ± 1.00	4.34 ± 1.16	5.06 ± 0.89*	4.53 ± 0.81	4.29 ± 0.96	4.99 ± 1.01	5.21 ± 1.02
LF, (ms)^2^	6.15 ± 0.73	5.94 ± 0.66	5.66 ± 0.81	5.53 ± 0.76	5.29 ± 0.78	4.88 ± 0.81*	4.65 ± 0.80	4.63 ± 0.69	4.38 ± 0.94	4.08 ± 0.92	5.21 ± 1.03	5.03 ± 1.00
VLF, (ms)^2^	6.22 ± 0.69	6.43 ± 0.71	6.02 ± 0.75	5.91 ± 0.77	5.55 ± 0.87	5.74 ± 0.72	5.31 ± 0.78	5.62 ± 0.78	5.48 ± 0.81	5.21 ± 0.70	5.70 ± 0.86	5.79 ± 0.83
TP, (ms)^2^	7.24 ± 0.57	7.34 ± 0.59	7.00 ± 0.56	6.85 ± 0.63	6.53 ± 0.67	6.47 ± 0.72	6.03 ± 0.75	6.38 ± 0.65	6.12 ± 0.71	5.89 ± 0.62	6.57 ± 0.80	6.59 ± 0.80
HRV-index	12.58 ± 3.01	12.90 ± 3.27	11.52 ± 3.03	11.02 ± 2.80	9.87 ± 2.76	9.66 ± 3.05	8.19 ± 2.68	9.26 ± 2.58	8.20 ± 1.90	7.79 ± 2.01	10.01 ± 3.18	10.15 ± 3.24
SDNN, ms	41.66 ± 11.17	44.45 ± 12.60	36.58 ± 9.96	34.48 ± 9.41	30.35 ± 9.80	30.06 ± 10.32	24.47 ± 9.64	28.58 ± 9.46	24.65 ± 7.97	22.34 ± 7.57	31.31 ± 11.69	32.07 ± 12.27
pNN50,%	37.80 ± 13.13	39.69 ± 12.97	32.45 ± 12.73	29.96 ± 13.04	24.25 ± 13.72	23.66 ± 13.34	14.98 ± 13.12	21.00 ± 13.07	15.47 ± 11.05	12.40 ± 10.91	24.69 ± 15.48	25.48 ± 15.46

Age is divided into groups of 20 years (19–29 years), 30 years (30–39 years), 40 years (40–49 years), 50 years (50–59 years), and 60 years (60–69 years). Data are presented as the number of subjects or mean ± SD values, as indicated. P-values are derived using the independent t-test considering gender differences (men versus women) in each age group. *P < 0.05, **P < 0.01.

**FIGURE 3:**
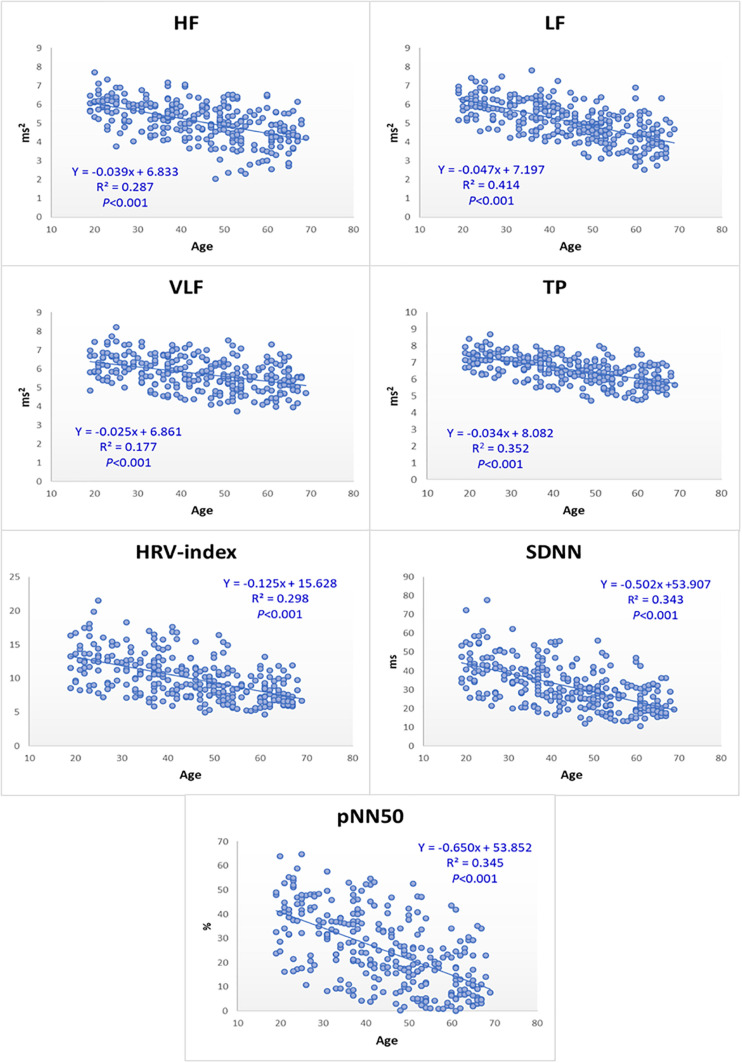
HRV indices decrease gradually with age in healthy Korean groups. HF, LF, VLF, and TP were expressed as the natural log-scaled power (ms^2^) of each frequency range; HR as beats per minute (bpm); SDNN as the standard deviation (ms) of heart rate variability; and pNN50 as the proportion (%) derived by dividing NN50 by the total number of NN intervals. Only HRV-index is unitless. For all HRV indices, the higher indices, the younger and healthier ANS.

The values of the HRV indices (HF, LF, VLF, TP, SDNN, HRV-index, and pNN50) have clearly shown a decreasing trend with age in healthy Korean groups, similar to that in the Western population, as shown in the Figure 3 (*P* < 0.001 for all indices). HF values decreased linearly with age (*Y* = −0.039x + 6.833, *R*^2^ = 0.287), as did LF (*Y* = −0.047x + 7.197, *R*^2^ = 0.414), VLF (*Y* = −0.025x + 6.861, *R*^2^ = 0.177), TP (*Y* = −0.034x + 8.082, *R*^2^ = 0.352), HRV-index (*Y* = −0.125x + 15.628, *R*^2^ = 0.298), SDNN (*Y* = −0.502x + 53.907, *R*^2^ = 0.343), and pNN50 (*Y* = −0.650x + 53.852, *R*^2^ = 0.345).

Table 1 shows gender differences in HRV parameters. All HRV indices had no significant gender differences except in 2 cases. Women had a higher HF value than men in the age group of 50 years (5.06 ± 0.89 versus 4.34 ± 1.16, *P* < 0.05). The LF value in the age group of 40 years was lower in women than in men (4.88 ± 0.81 versus 5.29 ± 0.78, *P* < 0.05). The gender difference in age was not significantly different except that there were older women than men in the age group of 50 years. The number of people by age group was quite similar, and the number of men and women depending on the age group was also very similar (27 versus 27 in 20 years, 28 versus 31 in 30 years, 30 versus 30 in 40 years, 29 versus 29 in 50 years, 30 versus 28 in 60 years; 144 men versus 147 women in total).

## Discussion

In this study, we have presented a linear regression model of various HRV indices with the age of healthy Asians and accordingly provided reference ranges for Asian women and men depending on the age groups. HRV clearly showed a decrease in HF, LF, VLF, TP, SDNN, HRV-index, and pNN50. Each index had a significant age-related decline. This tendency is consistent with that in previous studies for Western people. Meersman and Stein reviewed that HF, LF, root mean square successive difference (rMSSD), and absolute number of adjacent normal RR intervals differing by >50 ms (sNN50) declined with advancing age (De Meersman and Stein, 2007). Voss et al. showed that HRV indices of HF, LF, SDNN, and pNN50, decreased drastically with age in 1,906 German subjects (Voss et al., 2015). Dietrich et al. (2006) found TP to decline clearly with age in 847 men. HRV parameters in Asian people also showed an aging tendency similar to that in Western people (Fukusaki et al., 2000; Baek et al., 2015), although a regression model of HRV with age was not provided yet.

In particular, this study has provided the clear linear regression model of various HRV indices, which is different from the previous ones that presented an age-related decline direction or shape of the indices. It may be utilized as estimating the functional age of each HRV parameter quantitatively. If the age estimated by this linear regression model is higher than the actual age (i.e., 10 years older), they will be classified as a group required ANS management. Therefore, autonomic degenerative disease may be prevented by recommending exercise and stress management, which can improve the group health.

Gender differences in HRV values have been shown in previous reports. Women had lower LF, TP, and SDNN than men in Switzerland, and female participants aged 25–49 years had lower HF and LF than age-matched male participants in Germany (Dietrich et al., 2006; Voss et al., 2015). However, LF and SDNN did not show gender differences in Japan, although women had higher HF than men (Fukusaki et al., 2000). The entire Korean group in this study did not show gender differences in all HRV indices of HF, LF, VLF, TP, SDNN, HRV-index, and pNN50, which showed a trend similar to the SDNN and LF of Japanese people. Interestingly, Umetani et al. (1998) revealed that HRV in young male participants was 53% higher than that in age-matched female participants, but the current study did not show gender differences in pNN50 with age, which has not previously been explored for the Asian population. This study was also the first to investigate VLF, TP, and HRV-index, for gender differences in the Asian population. The cause of the difference in HRV depending on the gender between Westerners and Asians should be included in future studies.

Heart rate variability can be used as an index to identify diabetic patients before the onset of autonomic neuropathy and to assess the risk of post-infarction mortality (Wolf et al., 1978; Ewing et al., 1985). Reduced HRV can be a strong and independent predictor of mortality after acute myocardial infarction (Kleiger et al., 1987). It was also revealed that reduced VLF, HF, TP, and SDNN were significantly associated with all-cause mortality (Tsuji et al., 1994). HRV analysis can quantitatively evaluate LF and HF nerve activity in the heart, including autonomic balance (Saykrs, 1973; Akselrod et al., 1981; Pagani et al., 1986; Malliani et al., 1991; Malik et al., 1993; Reardon and Malik, 1996; Task Force of The European Society of Cardiology, and The North American Electrophysiology Society of Pacing and Electrophysiology, 1996; Dietrich et al., 2006; Maunder et al., 2006; Voss et al., 2015; Choi et al., 2017; Hirsch and Bishop, 2017). For instance, the smaller the SDNN, that is, the more constant the heartbeat interval, the lower the ability to regulate the vagus nerve, which acts as a pacemaker; the autonomic nervous system is easily unstable even in a small external stress situation, resulting in a decrease in adaptability or resistance. Usually, younger and healthier people tend to have a higher SDNN. With this background, it has been well established that loss of HRV occurs with aging (Pfeifer et al., 1983; De Meersman and Stein, 2007; Bishop et al., 2010; Simpson et al., 2014; Choi et al., 2019), which has been re-confirmed by this study even for the Asian population.

There are several limitations in the methods of this study. HRV can be ideally assessed in a person with BMI of 18.5–25 kg/m^2^. However, overweight individuals with BMI > 25 kg/m^2^ included in the assessment due to limited number of volunteers. In addition, we did not consider women menstrual cycle or daily alcohol consumption, although HRV is influenced by them (Bai et al., 2009; Quintana et al., 2013). Also, Individuals under treatment for diabetes or respiratory disorder were not excluded from the analysis. In application of HRV, the assessment of functional aging with HRV is accurate only when food, caffeinated drinks, nervous system drugs, and exercise have not been experienced 1 h before the measurement (Task Force of The European Society of Cardiology, and The North American Electrophysiology Society of Pacing and Electrophysiology, 1996). It is also difficult to assess subjects with unstable pulses and frequent arrhythmia owing to atrial fibrillation (Task Force of The European Society of Cardiology, and The North American Electrophysiology Society of Pacing and Electrophysiology, 1996). Lastly, the data would not be applied to Asian individuals from Americas and Europe because they have different dietary and lifestyle from Asians in Asia, which may affect their body condition.

Nevertheless, the distinct direction of HRV indices with age was shown in this study, which will be useful to prevent diseases related to the autonomic nervous system due to aging or degeneration of the autonomic nerve function in Asians. Moreover, it is expected to be more actively applied to quantify positive and negative changes before and after the treatment or therapy, such as stimuli, drugs, and exercise with sensitive levels.

## Conclusion

The tendency of HRV parameters was evaluated with increasing age for healthy Korean adults. Therefore, we have provided a linear regression model in which the HRV parameters decrease with advancing age in the Asian population (*P* < 0.001 for all models); HF, *Y* = −0.039x + 6.833; LF, *Y* = −0.047x + 7.197; VLF, *Y* = −0.025x + 6.861; TP, *Y* = −0.034x + 8.082; HRV-index, *Y* = −0.125x + 15.628; SDNN, *Y* = −0.502x + 53.907; pNN50, *Y* = −0.650x + 53.852. There was no significant gender difference in the HRV values. These distinct decline models of HRV indices with age, regardless of the gender, are expected to be useful in preventing diseases related to the autonomic nervous system by evaluating or tracking the autonomic functional deterioration in Asians.

## Data Availability Statement

All the data supporting the findings of this study are available from the corresponding author upon reasonable request.

## Ethics Statement

The studies involving human participants were reviewed and approved by the Asan Medical Center Institutional Review Board, South Korea (AMC-IRB-2007-0305). The patients/participants provided their written informed consent to participate in this study.

## Author Contributions

JC conceived and designed the manuscript, and contributed to data acquisition. M-GP wrote the first draft of this manuscript. JC and M-GP reviewed this manuscript. JC and WC analyzed and interpreted the data. All authors read and approved the final manuscript.

## Conflict of Interest

The authors declare that the research was conducted in the absence of any commercial or financial relationships that could be construed as a potential conflict of interest.
